# FDG-PET/CT and Multimodal Machine Learning Model Prediction of Pathological Complete Response to Neoadjuvant Chemotherapy in Triple-Negative Breast Cancer

**DOI:** 10.3390/cancers17071249

**Published:** 2025-04-07

**Authors:** David Groheux, Loïc Ferrer, Jennifer Vargas, Antoine Martineau, Adrien Borgel, Luis Teixeira, Philippe Menu, Philippe Bertheau, Olivier Gallinato, Thierry Colin, Jacqueline Lehmann-Che

**Affiliations:** 1 Department of Nuclear Medicine, AP-HP, Saint-Louis Hospital, F-75010 Paris, France; antoine.martineau@aphp.fr; 2 Université Paris Cité, Inserm, Institut de Recherche Saint Louis (IRSL), F-75010 Paris, France; adrien.borgel@aphp.fr (A.B.); luis.teixeira@aphp.fr (L.T.); jacqueline.lehmann-che@aphp.fr (J.L.-C.); 3 SOPHiA GENETICS, F-33600 Pessac, France; lferrer@sophiagenetics.com (L.F.); jvargas@sophiagenetics.com (J.V.); ogallinato@sophiagenetics.com (O.G.); tcolin@sophiagenetics.com (T.C.); 4 Molecular Oncology Unit, AP-HP, Saint Louis Hospital, F-75010 Paris, France; 5 Breast Diseases Unit, AP-HP, Saint Louis Hospital, F-75010 Paris, France; 6 SOPHiA GENETICS, 1180 Rolle, Switzerland; pmenu@sophiagenetics.com; 7 Department of Pathology, AP-HP, Saint Louis Hospital, F-75010 Paris, France; philippe.bertheau@aphp.fr

**Keywords:** FDG-PET/CT, triple-negative breast cancer, neoadjuvant chemotherapy, artificial intelligence, machine learning, radiomics, metabolic response, pCR, prognosis

## Abstract

**Purpose:** Triple-negative breast cancer (TNBC) is a biologically and clinically heterogeneous disease, associated with poorer outcomes when compared with other subtypes of breast cancer. Neoadjuvant chemotherapy (NAC) is often given before surgery, and achieving a pathological complete response (pCR) has been associated with patient outcomes. There is thus strong clinical interest in the ability to accurately predict pCR status using baseline data. **Materials and Methods:** A cohort of 57 TNBC patients who underwent FDG-PET/CT before NAC was analyzed to develop a machine learning (ML) algorithm predictive of pCR. A total of 241 predictors were collected for each patient: 11 clinical features, 11 histopathological features, 13 genomic features, and 206 PET features, including 195 radiomic features. The optimization criterion was the area under the ROC curve (AUC). Event-free survival (EFS) was estimated using the Kaplan–Meier method. **Results**: The best ML algorithm reached an AUC of 0.82. The features with the highest weight in the algorithm were a mix of PET (including radiomics), histopathological, genomic, and clinical features, highlighting the importance of truly multimodal analysis. Patients with predicted pCR tended to have a longer EFS than patients with predicted non-pCR, even though this difference was not significant, probably due to the small sample size and few events observed (*p* = 0.09). **Conclusions:** This study suggests that ML applied to baseline multimodal data can help predict pCR status after NAC for TNBC patients and may identify correlations with long-term outcomes. Patients predicted as non-pCR may benefit from concomitant treatment with immunotherapy or dose intensification.

## Simple Summary

Triple-negative breast cancer is a heterogeneous disease associated with poor outcomes. Often treated with neoadjuvant chemotherapy, achieving pCR at the end of treatment is a goal and predicts patient survival. But predicting pCR from the outset could be of clinical interest and avoid the administration of ineffective treatments. Here we present a proof-of-concept study of a multimodal machine learning algorithm incorporating PET (including radiomics), histopathological, genomic, and clinical features. The algorithm developed can predict pCR in triple negative breast cancers with an AUC of 0,82 and show a tendency to correlate with long-term outcomes. This early prediction of response to chemotherapy could lead to propose more personalized treatment.

## 1. Introduction

Many patients with stage II–III breast cancer (BC) receive neoadjuvant chemotherapy (NAC) [1]. This strategy allows more patients to undergo breast-conserving surgery (BCS) and increases the chances of surgery in patients with primary inoperable disease; it also provides information on the efficacy of chemotherapy [2]. Pathological complete response (pCR) after NAC is a strong predictor of favorable outcomes, especially in aggressive breast cancer subtypes such as human epidermal growth factor receptor 2-positive (HER2+) breast cancer and triple-negative breast cancer (TNBC; lacking estrogen receptor (ER), progesterone receptor (PR), and HER2 overexpression) [3,4]. However, the pathological response is only known at the end of NAC, so the earlier detection of treatment response would lead to rapid treatment adaptation to increase the pCR rate in non-responders [5]. Positron emission tomography/computed tomography (PET/CT) with ^18^F-fluorodeoxyglucose (FDG) has shown potential to detect residual disease early (after one or two cycles of NAC) and also to predict poor outcomes in TNBC patients [6,7,8,9]. The PET image-derived parameter used in most studies is the decrease in the maximum standard uptake value (SUV_max_) under therapy. Unfortunately, the percentage change in FDG uptake, which was previously considered discriminant, varies dramatically across studies, preventing the translation of this technique to clinical practice. Moreover, this method requires two sequential PET scans to measure an early change in the standardized uptake value (ΔSUV) [10,11,12] and this is not typically a standard of care [1,13].

Various clinical, histopathological, and biological breast cancer characteristics assessed before treatment are well known as prognostic factors. In particular, high-grade tumors are more proliferative and aggressive than low-grade tumors and more prone to respond to chemotherapy. However, tumor grade alone has some limitations in predicting response to treatment, especially in the case of TNBC, where most of these tumors are of a high grade. Thus, the genomic grade index (GGI) was developed to improve BC grading and its prognostic value [14], and we demonstrated that the prediction of response to NAC increased when baseline SUV_max_ was combined with the GGI in a previous study on TNBC patients [15]. By optimizing data from medical imaging, radiomics has also shown the ability to predict response to neoadjuvant chemotherapy for breast cancer [16,17,18]. Artificial intelligence (AI) techniques with machine learning (ML) and deep learning (DL), a subset of ML, have also recently shown the ability to improve breast imaging performance [19], including therapeutic prediction through PET imaging [20,21]. In this context, the present study’s main objective was to evaluate the value of a multimodal machine learning-based algorithm predictive of pCR, combining multiple parameters contributing to the prediction of response, such as clinical, histopathological, genomic, and PET imaging (including radiomics) data before treatment. This should make it possible to predict the response to NAC on the basis of data available at diagnosis for any patient undergoing treatment for breast cancer.

## 2. Patients and Methods

### 2.1. Study Design

The present study was designed to evaluate the predictive value of a combination of parameters based on clinical data (e.g., age, family history of breast cancer, clinical T-stage, clinical N-stage, unifocal or multifocal tumor), histopathological findings (grade, Ki-67 IHC expression), molecular markers measured on pretreatment biopsy (e.g., GGIr, or isolated components of the GGIr: CDC2, CDC20, KPNA2, and MYBL2 gene expression), and FDG-PET/CT imaging features (e.g., tumor SUV_max_, lymph node SUV_max_, metabolic tumor volume, radiomic features…). All features were collected in a large database to be processed by a machine learning algorithm.

Patients meeting the eligibility criteria had stage II–III triple-negative breast cancer (TNBC), were scheduled for neoadjuvant chemotherapy, and had a baseline PET. Patients with distant metastases and patients with bilateral cancer were not included.

We first determined the ability of these parameters, alone or in combination, to predict pCR (primary endpoint). Then, we tested if the predicted pCR was a surrogate marker of each patient’s outcome (secondary endpoint).

The Institutional Review Board approved the study and stated that no informed consent was needed, considering the non-interventional design of this retrospective analysis (IRB # 00003835, French ethics committee Paris-Saint-Louis, # 2013-27NICB; NCT02600442).

### 2.2. Histopathological Features and Gene Expression Profiling

Breast cancers were diagnosed based on an ultrasound-guided core-needle biopsy. An experienced pathologist determined tumor type and histological grade using the modified Scarff–Bloom–Richardson (SBR) grading system for invasive carcinoma.

Tumors were defined as triple negative based on immuno-histochemical staining using specific antibodies and an automated immunostainer (Ventana XT; Tucson, AZ, USA). Tumors were considered ER and PR negative if less than 10% of tumor cells expressed ER and PR. HER2 was over-expressed if HER2 immunostaining was uniform with intense membrane staining of >30% of invasive tumor cells, following the recommendations for the period of analysis.

Ki67 score was analyzed by immunohistochemistry using an MIB-1 antibody (Dako, Glostrup, Denmark) and quantified automatically by the image analysis software Hamamatsu NDP Analyze software in partnership with Visiopharm. The threshold for Ki67 positivity was 14% stained cells, whatever the staining intensity [22].

Total RNA extracted from frozen biopsy was used for molecular analysis. TP53 functional status was determined using a highly efficient yeast functional assay (FASAY) as previously described [23].

Gene expression analysis of Ki67, CDC2, CDC20, KPNA2, and MYBL2 was performed by RT quantitative PCR. GGIr scores were obtained by combining the expression of the 4 genes (CDC2, CDC20, KPNA2, and MYBL2), covering all cell cycle phases, as described previously [24]. We analyzed the predictive value of Ki67 mRNA expression and of the reduced genomic grade index (GGIr) as a continuous variable for the association with pCR.

### 2.3. FDG-PET/CT Image Acquisition

Patients fasted for 6 h to achieve a blood glucose level less than 7 mmol/L. FDG (5 MBq/kg) was administered, and imaging (from the mid-thigh level to the base of the skull with the arms raised) started almost 60 min later. The Gemini XL PET/CT scanner (Philips Medical systems) was used. CT data were acquired first (120 kV; 100 mAs; no contrast-enhancement). PET emission data were acquired in a 3-dimensional mode with 2 min per bed position. The attenuation-corrected images were normalized for injected dose and body weight and subsequently converted into standardized uptake values (SUVs), defined as [tracer concentration (kBq/mL)]/[injected activity (kBq)/patient body weight (g)]. SUV_max_ was measured in the primary tumor and in the axillary lymph nodes if present.

### 2.4. Imaging Processing and Radiomic Features

**Figure 1:**
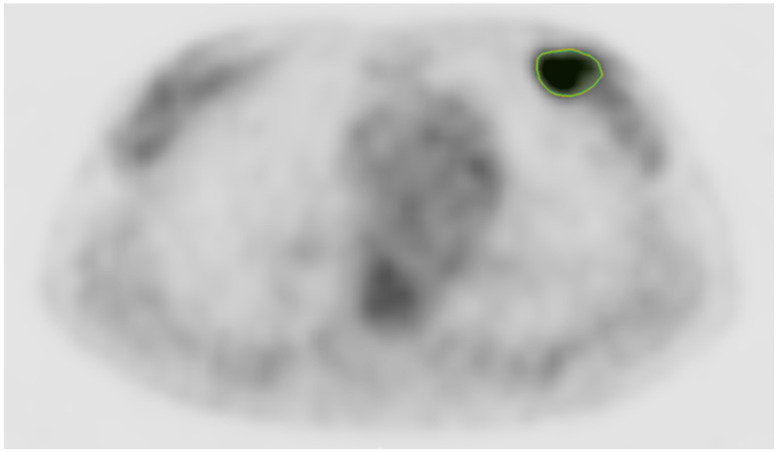
Example of 3D segmentation using a semi-automatic method based on 42% of the SUV_max_. The green contour delineates the segmented primary breast tumor lesion.

From the baseline PET/CT imaging data, the primary breast tumor of each patient was segmented in 3D through a semi-automatic segmentation method using 42% of the SUV_max_ (Figure 1). The segmentation was performed by an experimented nuclear physician using the SOPHiA DDMTM for Radiomics platform (Research Use Only; SOPHiA GENETICS SA; Rolle, Switzerland). Radiomics features describing the tumor through its size, shape, voxel intensity distribution, and texture were then extracted following the IBSI standards [25]. Metabolic tumor volume (MTV) was determined using the SOPHiA platform.

### 2.5. Neoadjuvant Chemotherapy Regimen

Some patients (the oldest treated) received EC-D (4 cycles of epirubicin 75 mg/m^2^ d1 plus cyclophosphamide 750 mg/m^2^ d1 administered every 3 weeks, followed by 4 cycles of docetaxel 100 mg/m^2^ d1 qw3). Patients from the more recent period received a dose-dense and dose-intense protocol, with epirubicin 75 mg/m^2^ d1 plus cyclophosphamide 1200 mg/m^2^ d1 every 2 weeks (SIM) for 6 cycles. After surgery, patients who received SIM chemotherapy received 3 cycles of docetaxel (75 mg/m^2^ d1 plus cyclophosphamide 750 mg/m^2^ d1) every 3 weeks.

### 2.6. Pathology Assessment, Follow-Up, and Event-Free Survival

Pathologic complete response (pCR) was defined as no evidence of residual invasive cancer in breast tissues and lymph nodes [4]. The absence of carcinoma in situ was not mandatory.

During neoadjuvant chemotherapy, patients underwent clinical examination every two cycles. After surgery, patients had follow-up visits every 4 months for two years, then twice yearly. Events included local, regional, or distant recurrences or death. Event-free survival (EFS) was defined as the period between the date of surgery and the date of the first event or the last follow-up.

### 2.7. Multimodal Data Aggregation

Clinical, histopathological, genomic, PET, and radiomic features were aggregated in a large database. Categorical features were one-hot encoded, and numerical features were standardized to achieve a null mean and unit variance. A batch-effect correction for genomic expression data was performed, inspired by the mean-only ComBat adjustment approach [26]. The association between each feature independently and pCR outcome was assessed using non-parametric tests (Wilcoxon rank-sum test for continuous covariates, Fisher’s exact test for binary covariates).

A hierarchical clustering method was applied to reduce the number of radiomic features, with a bootstrap approach to determine the optimal number of clusters given the stability of the partitions. Six groups of radiomic features were defined, and only one feature per group was selected. Relevant non-radiomic features were selected based on their completion rate (less than 50%) and univariable feature-outcome analyses combined with clinical expertise. A single imputation by chained equations was then carried out to handle missing values in predictors using the MICE algorithm with randomized decision trees and 10 iterations [27].

### 2.8. Machine Learning Model Development

Several machine learning algorithms were then trained, including logistic regression models (with either LASSO, Ridge, or Elastic-net regularization), binary decision trees, support vector machines (with linear kernel), and random forests. Due to the small cohort size, a nested leave-pair-out cross-validation (LPOCV) approach (60 random pairs in the inner resampling, 756 (all) random pairs in the outer resampling) was used to correctly estimate the predictive performance of the models and select the best one [28]. A grid search was applied with the area under the ROC curve (AUC) as the optimization criterion. The Additional Materials present the grid of hyperparameters that was explored for each model. The uncertainty of the estimated predictive metrics (95% confidence intervals) and the comparative performances obtained using various sets of data modalities (*p*-values) were quantified through 10,000-sample bootstrapping with outcome stratification over the pairs of patients used for the nested LPOCV.

The best ML model, according to the estimated predictive performances, was finally trained using a “standard” leave-pair-out cross-validation procedure. Global interpretability tools ensured the correct understanding, validation, and justification of the prediction model. In addition, Shapley additive explanations (SHAP) values were used to explain each patient-specific predicted probability of non-pCR.

EFS was estimated using the Kaplan–Meier method. The log-rank test was used to compare EFS among patients with predicted pCR vs. non-pCR using the best ML model.

All statistical analyses were performed in Python (version 3.8.0), using the scikit-learn (version 1.1.1) and lifelines (version 0.27.7) libraries.

## 3. Results

### 3.1. Patient Characteristics and Database Construction for Machine Learning

The study enrolled 57 patients with stage II or III TNBC treated in the neoadjuvant setting between 2008 and 2015 at the Saint Louis hospital. No patients had distant metastases on pretherapeutic PET/CT. Lymph node involvement was clinically apparent in 57.9% of patients (Table 1). Most tumors were of no specific histological type (52 breast carcinoma of non-specific type and 5 metaplastic carcinoma) and grade 3 (91.2%). At baseline, the median tumor SUV_max_ was 10 (min = 3; max = 31.4), and the median MTV was 7.9 cm^3^ (Table 1).

Eight patients received EC-D, while 49 were treated with the SIM protocol. Surgery was performed after NAC in all patients (28 breast-conserving surgeries and 28 mastectomies), except in one case showing clinical progression during neoadjuvant treatment.

**Figure 2:**
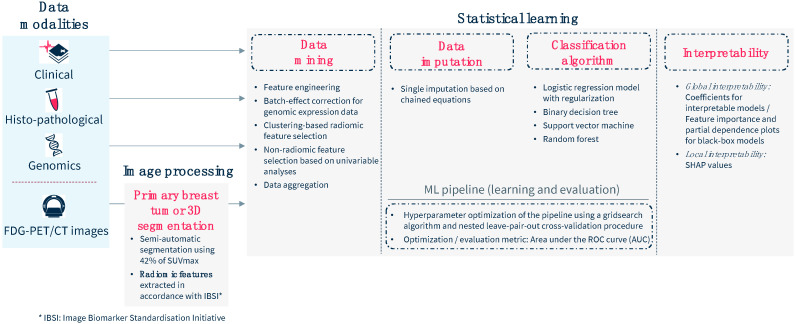
Outline of the multimodal analysis workflow for model development and evaluation.

The multimodal pretreatment data were aggregated, resulting in a total of 241 predictors collected for each patient (Figure 2): 11 clinical features, 11 histopathological features, 13 genomic features, and 206 PET features, including 195 radiomic features (20 describing the tumor size, 13 characterizing its shape, 76 describing its voxel intensity distribution, and 86 its texture; radiomic feature extraction is shown in the Supplementary Materials).

### 3.2. Association Between Main Features and pCR (Univariate Analysis)

More than half of the patients had non-pCR (36 of 57 patients; 63.2%). Table 2 shows the independent associations of main features with the pathological findings.

No significant relation between the chemotherapy regimen and pCR was observed, but the number of patients treated with EC-D was limited (pCR rate: 37.5% for the 8 patients treated with EC-D vs. 36.7% for the 49 patients who received SIM, *p* = 1.0).

We found no significant relation between tumor histology and pCR (*p* = 0.64) or between grade and pCR (*p* = 0.15); however, most patients had high-grade, invasive, non-specific-type carcinoma. The Ki67 score measured by IHC and the gene expression of Ki67 were not associated with pCR. During gene expression profiling, GGIr (and its components) was associated with the pathological response (Table 2). Pathological complete response (pCR) was more frequent in T1–T2 tumors than in T3–T4 tumors (*p* = 0.03). The absolute value of the SUV measured at baseline PET was not significantly associated with pCR (*p* = 0.07); however, there was a trend for a higher SUV_max_ in the case of pCR (13.2 vs. 9.2).

### 3.3. Prediction of pCR with ML Algorithm

Of the 241 features collected for each patient, 17 features were selected for the final analysis (Table 2: features in bold): 3 clinical features (clinical T-stage, contraception, and family history of breast cancer), 2 histopathological features (mitoses count, Ki-67 score determined by automate), 3 molecular features (Ki-67 mRNA expression, TP53 mutational status evaluated by functional assay and GGIr), 3 PET non-radiomic features (tumor SUV_max_, lymph node SUV_max_, and MTV), and 6 radiomic parameters (morphological sphericity, intensity skewness, discretized intensity uniformity, GLCM contrast, GLDZM large distance low gray-level emphasis, and NGLDM low dependence low gray-level emphasis). The pCR prediction performance of each machine learning model was estimated according to several sets of the multimodal predictors (Table 3). The best ML predictive model was a support vector machine (SVM) algorithm with a linear kernel (Table 3), and the best predictive results were achieved using the aggregation of clinical data, histopathological and molecular features, and PET data, including radiomic features (Table 3). Considering the SVM algorithm, the AUC was 0.63 (95% CI = 0.51–0.73) for clinical + histopathological + PET non-radiomic data (first set), 0.70 (0.60–0.80) after adding molecular data (second set), and finally 0.82 (0.74–0.90) after also including the whole set of radiomic features. The estimated AUC was significantly greater when considering the whole set of multimodal data than using the first and second sets of data modalities mentioned above, with *p* = 0.001 (whole set of data vs. first set) and *p* = 0.03 (whole set of data vs. second set), respectively. In contrast to the value of 0.82 for SVM, the AUC was 0.67 for the decision tree algorithm, 0.65 for the random forest algorithm and 0.64 for the logit algorithm (Table 3).

**Figure 3:**
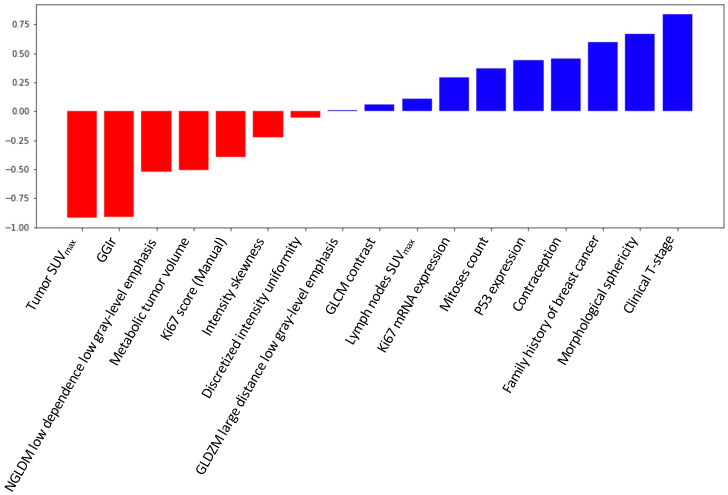
Diagram representing the coefficients of the support vector machine ML model.

Figure 3 shows the estimated coefficients of each feature in the linear SVM model. This model enables the direct interpretation of coefficients, as they represent feature weights. Tumor SUV_max_, GGIr, and clinical T-stage were found as the three most important predictors. Four of the seven most important features were derived from PET imaging (baseline tumor SUV_max_, MTV, and two radiomic features: morphological sphericity and NGLDM low dependence low gray-level emphasis).

### 3.4. Event-Free Survival

**Figure 4:**
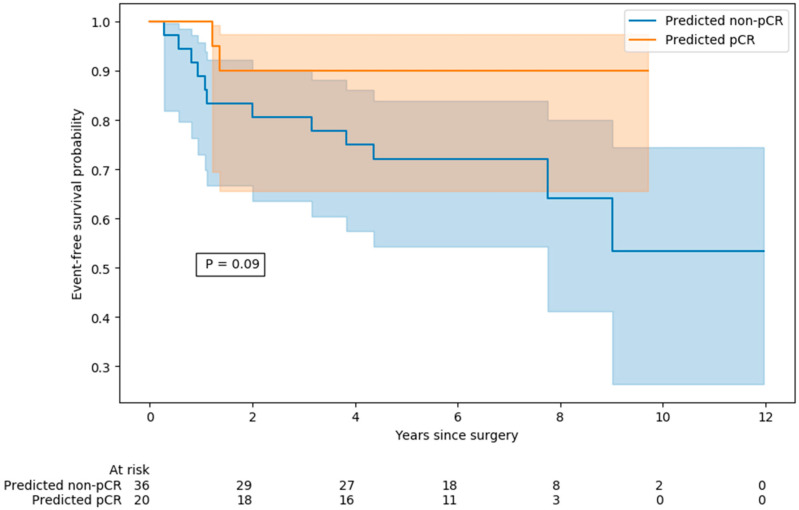
Kaplan–Meier estimates of event-free survival stratified according to predicted pathological complete response status by the support vector machine model, with *p*-values estimated by the log-rank test.

Among the 56 patients included for EFS analysis, 15 patients relapsed during the follow-up period. As shown in Figure 4, patients with predicted pCR tended to have a longer EFS than patients with predicted non-pCR, even though this difference was not significant, probably due to the small sample size and few events observed (*p* = 0.09).

## 4. Discussion

In a homogenous series of 57 TNBC patients, although baseline PET SUV_max_ alone was not predictive of pathological response after neoadjuvant chemotherapy, a dataset of 17 pretherapeutic features (mixing clinical, histopathological, molecular, and pretreatment PET findings) processed with a machine learning algorithm was highly predictive of pCR (AUC = 0.82 (95% CI = 0.74–0.90) with the SVM algorithm). In our previous study on TNBC patients, we observed that tumor baseline SUV_max_ value combined with some molecular features was predictive of pCR [15], but with limited performance (AUC = 0.76 for baseline SUV_max_ + GGI) [15]. The change in FDG uptake between the baseline PET and an interim PET performed after two cycles of NAC (ΔSUV_max_) was related to the pCR rate with a higher accuracy (AUC = 0.81) [15]. In the present study, the interim PET was not used. We focused on a panel of features, all determined at baseline, and the support vector machine (SVM) algorithm predicted the pCR with an AUC of 0.82. Among various prediction models tested here (logistic regression with regularization methods, binary decision tree, random forest, and support vector machine (linear kernel) techniques, the SVM algorithm had a better performance.

Machine learning and deep learning are of growing interest in radiology and nuclear medicine. Different studies have evaluated the usefulness of AI in predicting response to breast cancer treatment and patient outcomes before starting the treatment itself. A recent meta-analysis reports the feasibility of such non-invasive radiomic approaches but highlights the heterogeneity of the studies published [17]. This type of approach was also used in nuclear medicine, albeit in a more limited way, in small breast cancer cohorts without focusing on cancer subtypes of particular interest, such as TNBC. Accordingly, in 56 breast cancer patients, the AUC for predicting a histopathological response after NAC improved after deep learning using a convolutional neural network (CNN) [20]. Different breast cancer subtypes were included, and subgroup analysis revealed that the datasets were not predictive in the triple-negative group [20]. Only eight patients had TNBC, and only four parameters were analyzed (three PET features and one MRI feature). In another study about FDG-PET/CT imaging and AI, a CNN model was also useful for predicted pCR (accuracy of 84.79%), but 31 patients with breast cancer of mixed subtypes were included, and only 3 had TNBC [29]. A more recent study on 73 breast cancer patients evaluated individual or combined FDG-PET/MRI radiomic features [21]. The pCR prediction accuracy was high in the entire cohort (AUC = 0.8) and was the best for HR+ tumors (*n* = 52, AUC = 0.94). For the 19 TNBC cases included in the study, the prediction accuracy was also high (AUC = 0.92), but with no impact of the addition of FDG-PET to MRI features. Moreover, a few studies combined not only imaging but also pathological or molecular parameters to improve the prediction [8,15,30], paving the way for combined predictive biomarker strategies. This promising approach using the multimodal integration of metabolic, pathological, and clinical parameters has not yet been fully explored and can now benefit from the incorporation of AI.

In our study focusing on 57 TNBC patients, the developed ML algorithm could predict pCR using a dataset of 17 features. The best result was obtained using the aggregation of clinical, histopathological, genomic, and PET features, highlighting the importance of a truly multimodal analysis. Withdrawing a specific data modality (e.g., radiomic features or genomic data) led to a decrease of almost 10% in the AUC (Table 3). PET features, including radiomics, were of importance in the model.

Four of the seven most important features were derived from PET imaging (baseline tumor SUV_max_, MTV, and two radiomic features: morphological sphericity and NGLDM low dependence low gray-level emphasis). These features were complementary, describing the tumor through its size, its shape, its voxel intensity distribution, and its texture.

Some of these criteria probably define triple-negative tumors well, which are aggressive tumors that can sometimes proliferate rapidly and heterogeneously, with areas of necrosis [31]. Optimal characteristics would likely be different for other molecular subtypes, including texture parameters, indicating the utility of developing specific predictive criteria for each of the molecular subgroups of breast cancer. Previously, in 143 consecutive estrogen receptor-positive/HER-negative (ER+/HER2−) breast cancer patients, we found a significant association between patient outcome and selected PET parameters measured at baseline: tumor SUV_max_, MTV, total lesion glycolysis, and entropy [32]. Other teams have also evaluated the ability of radiomic parameters to predict response in patients treated with neoadjuvant chemotherapy for breast cancer, but these works have involved tumors with different molecular subgroups [16,33,34,35]. In a study of 79 patients with BC of different subtypes, the relationship between pretreatment PET parameters, including radiomic features, and pCR to NAC was analyzed by multiple logistic regression models [35]. All models showed that the molecular subtype of the tumor was the main predictor [35].

Contrary to previously published studies, focusing on a homogeneous group of breast cancers is a strength of our study. Moreover, this study is also based on an extended follow-up, as the patients were treated between 2008 and 2015, and the follow-up period continued for 12 years after surgery (Figure 4). The recurrence of triple-negative breast cancer usually occurs early, within 2–3 years of the completion of neoadjuvant chemotherapy [3]. As shown in Figure 4, patients with predicted pCR based on the multimodal algorithm tended to have longer event-free survival than patients with predicted non-pCR, even though this difference was not significant, probably due to the small sample size and few events observed. Our observation is, however, in agreement with the literature data, where pCR is a strong predictor of survival in TNBC [4]. In 11,955 patients, pCR was associated with improved EFS, and the association between pCR and long-term outcomes was strongest in patients with TNBC and in those with HER2+/ER- tumors who received trastuzumab [4]. In two multicenter prospective studies, FDG-PET findings were used to change neoadjuvant treatment early in patients with HER2+ BC [5,36]. In the randomized phase 2 AVATAXHER study, the addition of bevacizumab after two cycles of neoadjuvant treatment in patients predicted to be poor responders based on FDG-PET/CT led to an increase in the pCR rate [5]. The PHERGAIN trial investigated a chemotherapy-free treatment approach based on a dual HER2 blockade with trastuzumab and pertuzumab, with treatment decisions made based on early response on FDG-PET during neoadjuvant therapy and then further adapted according to pathology findings at surgery [36]. With this strategy, the 3-year invasive disease-free survival was 94.8%, and about a third of patients could avoid chemotherapy [36]. Such approaches of reducing treatment in responding patients and/or increasing treatment in patients for whom early PET predicts the absence of pCR could be used in triple-negative breast cancer. The identification of patients not predicted to achieve pCR under standard chemotherapy could enable the modulation of the usual treatment with additional immunotherapy or dose intensification.

Our study has some limitations. The small number of patients and the single-institution design may limit generalizability but allowed for the establishment of a homogeneous cohort with extended follow up. Furthermore, given the limited sample size, the ML model was validated and trained exclusively on the same dataset. To offset these limitations, particular attention was paid to the methods used, such as the nested leave-one-pair-out cross-validation, which ensures both correct training and evaluation of the model’s performance. However, our results will have to be confirmed by an external validation in a large, multicenter population. Nevertheless, this study is a proof of concept for the potential value of multimodal models evaluating routinely available clinical, pathological, biological, and nuclear imaging parameters to predict response to neoadjuvant chemotherapy and guide the treatment regimen choice for patients.

## 5. Conclusions

In conclusion, our study suggests that machine learning applied to baseline multimodal data can help predict pCR status before the initiation of treatment in the neoadjuvant setting for TNBC patients and identify correlations with long-term outcomes. Identifying patients not predicted to achieve pCR under standard chemotherapy right from diagnosis could enable the modulation of the usual treatment with the addition of immunotherapy or dose intensification. These tools can allow for precision medicine and cost-effective treatment decisions, which should also be applied to chemotherapy regimens.

## Figures and Tables

**Table 1:** Overall characteristics of the 57 triple-negative breast cancer patients.

Patient Characteristics (N = 57)	Summary *
**Age in years**	54.0 (45.0, 64.0)
**Family history of breast cancer**	
No	46 (80.7)
Yes	10 (17.5)
Missing	1 (1.8)
Clinical T-stage **	
T1–T2	27 (47.4)
T3–T4	30 (52.6)
Clinical N-stage **	
N0	24 (42.1)
N+	33 (57.9)
**Histological type**	
Non-specific	52 (91.2)
Metaplastic	5 (8.8)
**Histological grade**	
Grade 1–2	5 (8.8)
Grade 3	52 (91.2)
**P53 mutation**	
Wild type	5 (8.8)
Mutated	52 (91.2)
**Ki-67 mRNA expression (×1000)**	533.1 (245.0, 781.6)
Missing	13
**GGIr † (×1000)**	391.1 (172.2, 598.4)
**Tumor SUV_max_**	10.0 (7.2, 14.8)
**Metabolic tumor volume (cm^3^)**	7.9 (3.8, 18.9)
**Chemotherapy regimen**	
EC-D	8 (14.0)
SIM	49 (86.0)
**Surgery**	
Breast-conserving surgery	28 (49.1)
Mastectomy	28 (49.1)
No surgery	1 (1.8)
**Pathological findings**	
pCR	21 (36.8)
non-pCR	36 (63.2)

* Continuous data: median (Q1: first quartile, Q3: third quartile); categorical data: amount (percentage). ** Clinical classification before FDG-PET/CT according to the eighth edition of the *AJCC Staging Manual*. † GGIr: reduced genomic grade index (calculated after batch-effect correction). EC-D, sequential regimen of four cycles of epirubicin 75 mg/m^2^ plus cyclophosphamide 750 mg/m^2^ followed by four courses of docetaxel 100 mg/m^2^; SIM, intensified regimen of epirubicin 75 mg/m^2^ plus cyclophosphamide 1200 mg/m^2^ for six cycles; DC, six cycles of docetaxel + cyclophosphamide; pCR, pathological complete response.

**Table 2:** Association between the main features and pCR.

Modality	Feature *	Non-pCR (N = 36, 63.2%)	pCR (N = 21, 36.8%)	*p*-Value
**Clinical**	Age in years (Q1, Q3)	54.0 (42.3, 63.0)	54.0 (49.0, 64.0)	0.73
	**Family history of breast cancer**			0.08
	No	27 (75.0)	19 (95.0)	
	Yes	9 (25.0)	1 (5.0)	
	Missing	0	1	
	**Contraception**			0.17
	No	14 (38.9)	12 (60)	
	Yes	22 (61.1)	8 (40)	
	Missing	0	1	
	Clinical T-stage *			**0.03**
	T1–T2	13 (36.1)	14 (66.7)	
	T3–T4	23 (63.9)	7 (33.3)	
	Clinical N-stage *			0.78
	N0	16 (44.4)	8 (38.1)	
	N+	20 (55.6)	13 (61.9)	
	Chemotherapy regimen			1.00
	EC-D	5 (13.9)	3 (14.2)	
	SIM	31 (86.1)	18 (85.8)	
	Surgery			0.27
	Breast-conserving surgery	15 (42.9)	13 (61.9)	
	Mastectomy	20 (57.1)	8 (38.1)	
	No surgery	1	0	
**Histopathological**	Histological type			0.64
	Non-specific	32 (88.9)	20 (95.2)	
	Metaplastic	4 (11.1)	1 (4.8)	
	Histological grade			0.15
	Grade 1–2	5 (13.9)	0 (0.0)	
	Grade 3	31 (86.1)	21 (100.0)	
	**Mitosis count**	20 (3, 30)	13.5 (3.7, 26.2)	0.83
	**Ki67 score (automated)**	35 (9, 64.5)	42 (31.2, 59.5)	0.35
**Gene expression profiling**	**Ki-67 mRNA expression** **Missing**	482.1 (280.8, 631.2)	579.0 (179.1, 862.6)	0.38
	**P53 mutation**			0.35
	Wild type	2 (5.6)	3 (14.3)	
	Mutated	34 (94.4)	18 (85.7)	
	CDC2 (×1000)	93.5 (53.4, 164.9)	202.5 (127.6, 289.1)	**0.02**
	CDC20 (×1000)	486.8 (214.4, 828.6)	1042.5 (505.2, 1353.5)	**0.04**
	KPNA2 (×1000)	251.8 (158.6, 339.9)	426.6 (272.9, 761.9)	**0.01**
	MYBL2 (×1000)	251.4 (129.0, 483.6)	421.9 (318.5, 636.9)	**0.01**
	GGIr ** (×1000)	322.5 (154.5, 440.5)	588.0 (376.6, 757.8)	**0.01**
**PET non-radiomic features**	**Tumor SUV_max_**	9.2 (7.0, 12.5)	13.2 (8.5, 20.1)	0.07
	**Lymph nodes SUV_max_**	1.4 (0, 9.6)	2.2 (0, 5.8)	0.95
	**Metabolic tumor volume (cm^3^)**	9.3 (4.9, 17.2)	6.6 (3.0, 23.6)	0.70
**Radiomics**	**Morphological sphericity (×1000)**	945.5 (911.5, 959.3)	935.0 (859.0, 959.0)	0.65
	**Intensity skewness (×1000)**	473.5 (321.3, 664.8)	610.0 (329.0, 816.0)	0.56
	**Discretized intensity uniformity (×1000)**	1.2 (0.9, 1.8)	1.9 (1.3, 2.4)	**0.05**
	**GLCM contrast**	31.0 (12.4, 46.6)	50.0 (18.0, 68.8)	0.12
	**GLDZM large distance low gray-level emphasis (×1000)**	2.2 (1.6, 4.5)	1.5 (1.1, 3.4)	0.14
	**NGLDM low dependence low gray-level emphasis (×10,000)**	0.8 (0.5, 1.1)	0.5 (0.4, 1.0)	0.14

* Clinical classification before FDG-PET/CT according to the eighth edition of the *AJCC Staging Manual*. EC-D, sequential regimen of four cycles of epirubicin 75 mg/m^2^ plus cyclophosphamide 750 mg/m^2^ followed by four courses of docetaxel 100 mg/m^2^; SIM, intensified regimen of epirubicin 75 mg/m^2^ plus cyclophosphamide 1200 mg/m^2^ for six cycles; DC, 6 cycles of docetaxel + cyclophosphamide; pCR, pathological complete response. ** GGIr: reduced genomic grade index (calculated after batch-effect correction); GGIr represents a combination of four genes (CDC2, CDC20, KPNA2, and MYBL2), covering all phases of the cell cycle. GLCM: gray-level co-occurrence matrix; GLDZM: gray-level distance zone matrix; NGLDM: neighboring gray-level dependence matrix. Continuous data: median (Q1: first quartile, Q3: third quartile). Categorical data: amount (percentage). *p*-values obtained from Wilcoxon rank-sum test for continuous data, Fisher’s exact test for categorical data. **Features in bold**: features selected for the final analysis. ***p*-value in bold**: significant *p*-value.

**Table 3:** Estimated predictive performances according to different sets of multimodal predictors and several machine learning algorithms.

Data Modalities		Model	AUC	Accuracy	Se	Sp	PPV	NPV
**1**	Clinical, histopathological, and PET non-radiomic features	**SVM**	0.63	0.53	0.57	0.48	0.60	0.40
		**Decision tree**	0.45	0.45	0.63	0.27	0.59	0.29
		**Random forest**	0.56	0.48	0.67	0.30	0.62	0.35
		**Logit**	0.49	0.44	0.15	0.73	0.50	0.34
**2**	**1 +** genomic data	**SVM**	0.70	0.61	0.58	0.63	0.73	0.47
		**Decision tree**	0.70	0.67	0.72	0.62	0.77	0.56
		**Random forest**	0.65	0.64	0.76	0.53	0.73	0.56
		**Logit**	0.65	0.61	0.61	0.60	0.72	0.47
**3**	**2 +** whole set of radiomic features	**SVM**	0.82	0.71	0.71	0.70	0.80	0.59
		**Decision tree**	0.67	0.65	0.74	0.56	0.74	0.55
		**Random forest**	0.65	0.54	0.74	0.35	0.66	0.44
		**Logit**	0.64	0.60	0.60	0.61	0.72	0.47

AUC: area under the ROC curve; Se: sensitivity; Sp: specificity; PPV: positive predictive value; NPV: negative predictive value; SVM: support vector machine (with linear kernel). The metrics were estimated using the nested leave-pair-out cross-validation.

## Data Availability

The data presented in this study are available in this article (and Supplementary Materials).

## Supplementary Materials

The following supporting information can be downloaded at: https://www.mdpi.com/article/10.3390/cancers17071249/s1, Figure S1: The image features pre-processing workflow; Figure S2: Image features families with dependence in preprocessed objects. Refs. [25,37,38] are cited in the Supplementary Materials.
