# Supplementary material for: FDG-PET/CT and Multimodal Machine Learning Model Prediction of Pathological Complete Response to Neoadjuvant Chemotherapy in Triple-Negative Breast Cancer
Source: Cancers (Basel). 2025 Apr 7;17(7):1249. doi: 10.3390/cancers17071249 (PMC11987901; doi:10.3390/cancers17071249)
Supplement: Supplementary file 1 [file cancers-17-01249-s001.zip › cancers-3537146-supplementary.pdf]

Additional Materials

RNA extraction and cDNA synthesis

Frozen sections of biopsies dedicated to RNA extraction were prepared by breast pathologists and were processed under RNAse-free conditions; RNA was extracted using Maxwell RSC simplyRNA Tissue Kit (Promega). First-strand cDNA synthesis was performed with 1µg total RNA using Superscript II Reverse Transcriptase (Invitrogen Corporation) in a final volume of 20 µL, as previously described [1].

Real-time RT-qPCR analysis

Quantitative PCR analysis was performed on 10 ng cDNA in duplicate. A 5 µL diluted sample of cDNA was added to 20 µL of the PCR mix. The thermal cycling conditions comprised an initial denaturation step at 95°C for 10 min, 45 cycles at 95°C for 15 sec, and annealing temperature, either 60°C or 65°C depending on the target, for 1 min.

All PCR reactions were performed using the QuantStudio™ 5 Dx Real-Time PCR Detection System (Applied Biosystems Inc., Forster City, USA). The PCR Core reagent kit was used for systems with Taqman probes (Eurogentec, Liège, Belgium). Predesigned TaqMan® Assay Reagents (ThermoFisher, Waltham, USA) were used for CDC20 (Assay ID Hs00426680_mH) and CDK1 (Assay ID Hs04177802_m1) mRNA expression. Primers and probes sequences used for MYBL2 and KPNA2 were previously described [2]. TATA Box binding protein (TBP) was used as endogenous reference genes. Target quantities were normalized to TBP mRNA expression.

Human breast luminal cancer cell lines T47D cDNA was used to generate 7 points standard curves for each gene. Target quantities were normalized to the reference genes and calibrated using the second point of each standard curve. Final results were expressed as N-fold differences in target gene expression relative to the reference genes and the calibrator and are expressed as: $2_{target}(Ct\ calibrator - Ct\ sample)/E_{reference\ gene}(Ct\ calibrator - Ct\ sample)$, where Ct is the cycle threshold. No reverse-Transcription Controls (NTC) were included in any batch of samples.

Radiomics feature extraction

The extraction process comprised two main steps, which were based upon the well-described recommendations of the image biomarker standardization initiative (IBSI) [3]:

1. Preprocessing of the image and the ROI,
2. Computation of the features.

The whole image features extraction process, using the dedicated SOPHiA GENETICS' library, was validated using phantoms and ground truths feature values provided by IBSI.

Image and ROI pre-processing

The overall pre-processing workflow is described in Figure S1, with all the possible configuration parameters displayed next to each step. However, only the steps relevant for the current study are detailed in this section. The parameter values representing the extraction configuration that were used for extraction are defined simultaneously.

Given an image and a segmentation stored as a 3D triangular mesh, the image was first cropped around the lesion with a margin of 10 voxels in each direction. This reduction in image dimensions significantly reduces computation time in subsequent steps, without inducing significant undesired edge effects.

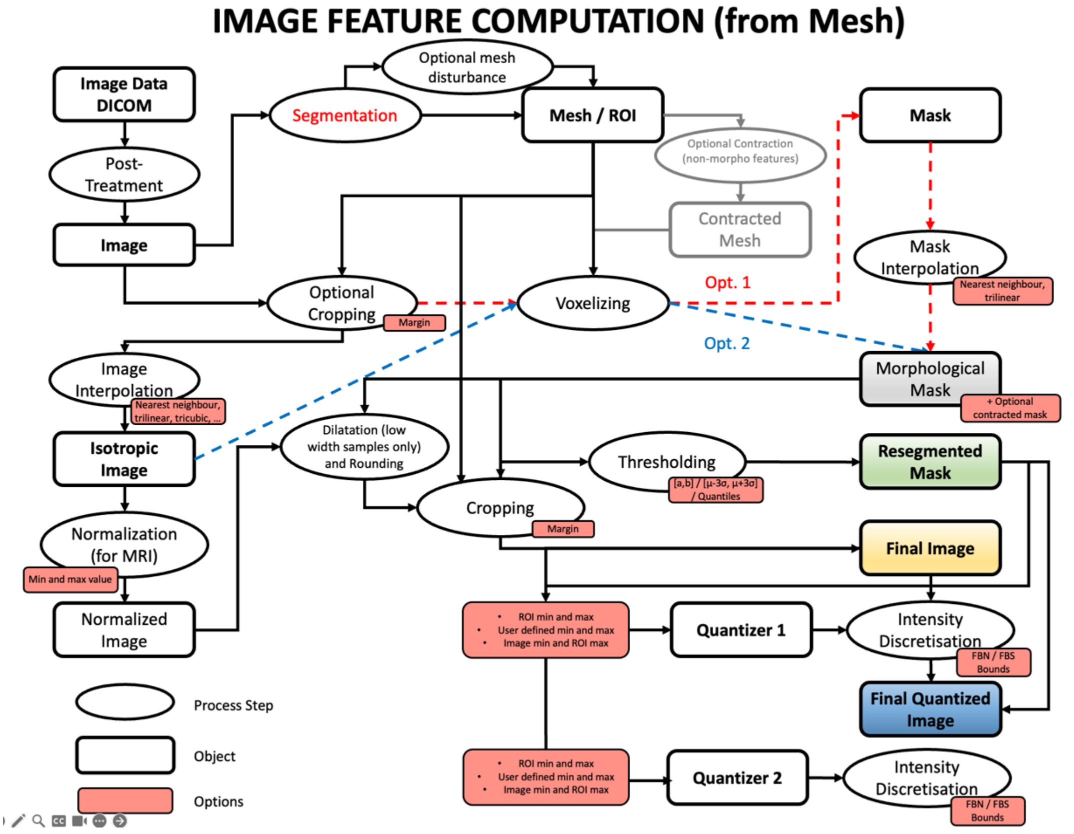

Figure S1. The image features pre-processing workflow.

The image was then resampled/interpolated to a 1x1x1mm³ voxel volume to achieve uniformity and comparability between all the images from which features are being extracted, and isotropy of the image response to textural features extraction that will be described later. Trilinear interpolation was used to assess the value of the resampled image voxels.

The ROI segmentation, still in the form of a 3D mesh, needed to be overlaid on the resampled image in a way that describes which voxels of the resampled image belong to the ROI. The most practical way to achieve this is to transform the mesh into a binary mask with the same grid characteristics as the resampled image. This transformation can be carried out by voxelizing the mesh on the image grid, which consists of the crossing number algorithm. It states that for any point inside the mesh, any line originating outside the polygon will cross the polygon an uneven number of times, thus enabling to detect voxel centers that lay within the closed polygon formed by the mesh.

No additional pre-processing steps induce edge-effects, meaning that a second cropping operation with a margin of 5 voxels in each direction was performed to reduce the image dimensions and allow for timesaving.

No re-segmentation or thresholding step was then applied to the morphological mask.

Finally, discretization or quantization of image intensities inside the ROI was required to make the calculation of texture features tractable and possessing noise-suppressing properties. The *Fixed Bin Size* method was used, with a bin width set to 0.1.

Image features computation

Image features, as defined by the IBSI, are divided into 4 main families: morphological, intensity-based, quantized intensity-based and textural features. Figure S2 shows a more detailed grouping of the approximately 200 features into families with the preprocessed objects required for computation.

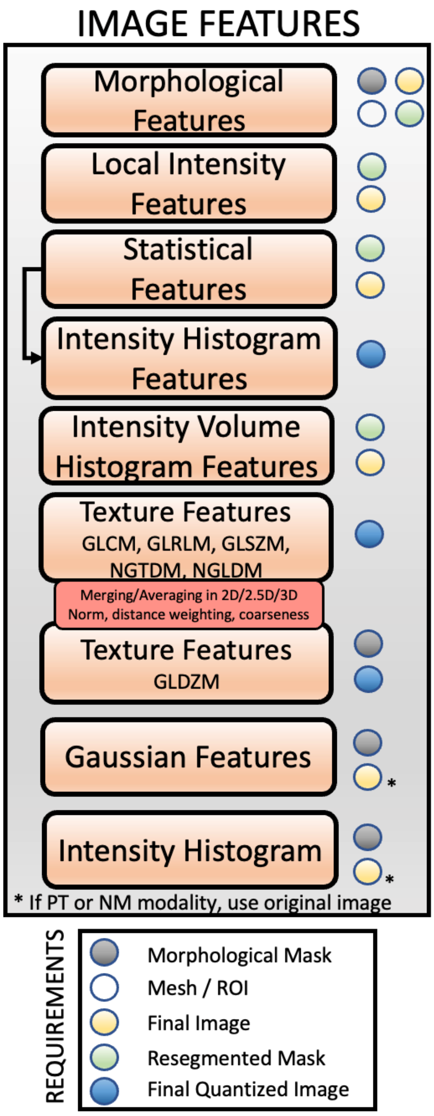

Figure S2. Image features families with dependence in preprocessed objects.

- *Morphological features*

Morphological features describe geometric aspects of the ROI, mainly based on area and volume of the mask and mesh representations of the ROI.

- *Intensity-based features*

This family is comprised of:

- Statistical features describing the overall distribution intensity within the ROI,
- Local intensity features which rely on the averaging of voxel intensities within a defined neighborhood around center voxels.

- *Quantized-intensity-based features*

Statistical features on a discretized representation of the grey level distribution. This family is comprised of:

- *Intensity histogram features*: an intensity histogram is generated by discretizing the original intensity distribution into grey level bins. Each bin of the histogram contains the proportion of voxels of grey level i. Statistical features similar to the intensity-based statistical features are then computed,
- *Intensity-volume histogram features*: describe the relationship between discretized intensity i and the fraction of the volume containing at least intensity i.

- *Textural features*

Describe the relationship between neighboring discretized voxels. This family is comprised of the following:

- *Grey level co-occurrence-based features*: the grey level co-occurrence matrix (GLCM) is a matrix that expresses how combinations of grey levels of neighboring voxels are distributed along one of the image directions,
- *Grey level run length-based features*: a run length is the length of a consecutive sequence of voxels with the same grey level in a given direction. The grey level run length matrix (GLRLM) contains the occurrences of runs with length j for a grey level i,
- *Grey level size zone-based features*: The grey level size zone matrix (GLSZM) counts the number of groups (or zones) of voxels of same grey level.
- *Grey level distance zone-based features*: The grey level distance zone matrix (GLDZM) measures the distances of groups (or zones) of voxels of same grey level to the boundary of the ROI,
- *Neighborhood grey tone difference-based features*: an alternative to the GLCM features,
- *Neighboring grey level dependence-based features*: an alternative to the GLCM features.

- *Additional features*

In addition to the IBSI features, we added some custom features:

- *Gaussian features:* decomposition of the intensity distribution histogram into an optimal number of gaussians.

Explored grid of models' hyperparameters

For each model, the optimal hyperparameters were found using a grid search procedure in a leave-pair-out cross-validation. Other parameters were set with default values by the Python library scikit-learn.

ML model	Hyperparameter	Explored space	Optimal value (for the full multimodal model)
Logistic regression	Penalty	L1	L1
	C	{0.01, 0.1, 0.2}	0.2
Binary decision tree	Max depth	{1, 3, 5, 7, 9}	3
Random forest	N estimators	500	500
	Max depth	{1, 4, 7, 10}	4
Support vector machine	Kernel	Linear	Linear
	C	{0.001, 0.01, 0.1, 0.5, 1}	0.5

References

1. Hamy, A.S.; Bieche, I.; Lehmann-Che, J.; Scott, V.; Bertheau, P.; Guinebretière, J.M.; Matthieu, M.C.; Sigal-Zafrani, B.; Tembo, O.; Marty, M.; et al. BIRC5 (Survivin): A Pejorative Prognostic Marker in Stage II/III Breast Cancer with No Response to Neoadjuvant Chemotherapy. *Breast Cancer Res. Treat.* **2016**, *159*, 499–511, doi:10.1007/s10549-016-3961-2.
2. de Cremoux, P.; Biard, L.; Poirot, B.; Bertheau, P.; Teixeira, L.; Lehmann-Che, J.; Bouhidel, F.A.; Merlet, P.; Espié, M.; Resche-Rigon, M.; et al. 18FDG-PET/CT and Molecular Markers to Predict Response to Neoadjuvant Chemotherapy and Outcome in HER2-Negative Advanced Luminal Breast Cancers Patients. *Oncotarget* **2018**, *9*, 16343–16353, doi:10.18632/oncotarget.24674.
3. Zwanenburg, A.; Vallières M.; Abdalah M.A.; Aerts H.J.W.L.; Andrearczyk V.; Apte A.; Ashrafinia S.; Bakas S.; Beukinga R.J.; Boellaard R.; et al. The image biomarker standardization initiative: standardized quantitative radiomics for high-throughput image-based phenotyping. *Radiology.* **2020**, *295.2*, 328–338, doi:10.1148/radiol.2020191145.
